# Using stochastic cell division and death to probe minimal units of cellular replication

**DOI:** 10.1088/1367-2630/aab197

**Published:** 2018-03-29

**Authors:** Savita Chib, Suman Das, Soumya Venkatesan, Aswin Sai Narain Seshasayee, Mukund Thattai

**Affiliations:** 1 Simons Centre for the Study of Living Machines, National Centre for Biological Sciences, TIFR, Bangalore, India; 2 Birla Institute of Technology and Science, Pilani, India; thattai@ncbs.res.in

**Keywords:** self replication, stochastic models, bacterial growth laws, antibiotic resistance

## Abstract

The invariant cell initiation mass measured in bacterial growth experiments has been interpreted as a minimal unit of cellular replication. Here we argue that the existence of such minimal units induces a coupling between the rates of stochastic cell division and death. To probe this coupling we tracked live and dead cells in *Escherichia coli* populations treated with a ribosome-targeting antibiotic. We find that the growth exponent from macroscopic cell growth or decay measurements can be represented as the difference of microscopic first-order cell division and death rates. The boundary between cell growth and decay, at which the number of live cells remains constant over time, occurs at the minimal inhibitory concentration (MIC) of the antibiotic. This state appears macroscopically static but is microscopically dynamic: division and death rates exactly cancel at MIC but each is remarkably high, reaching 60% of the antibiotic-free division rate. A stochastic model of cells as collections of minimal replicating units we term ‘widgets’ reproduces both steady-state and transient features of our experiments. Sub-cellular fluctuations of widget numbers stochastically drive each new daughter cell to one of two alternate fates, division or death. First-order division or death rates emerge as eigenvalues of a stationary Markov process, and can be expressed in terms of the widget’s molecular properties. High division and death rates at MIC arise due to low mean and high relative fluctuations of widget number. Isolating cells at the threshold of irreversible death might allow molecular characterization of this minimal replication unit.

## Introduction

There are many approaches to define a ‘minimal cell’. Some attempt to construct protocells out of elementary molecules and chemical processes [1, 2]. Others start with a complex cell and reduce it to an essential core [3]. A third fruitful approach uses natural patterns of cell growth to infer basic requirements for cellular replication [4–6]. Campbell [5] realized that exponentially growing cellular populations were in a state of ‘balanced growth’: the chemical composition of a daughter cell immediately after division was invariant from one generation to the next, leading to a well-defined and constant doubling time. The specific dependence of the exponential growth rate (the exponent of the cell density versus time curve) on nutrient or antibiotic concentrations can be summarized as ‘growth laws’ [7]. Models of bacteria as autocatalytic chemical reactors accurately capture many mathematical features of these growth laws [8–11]. Combining such models with bacterial growth measurements, Jun and colleagues [11] have demonstrated an invariant cell initiation mass which they interpret as a minimal unit of cellular replication.

Studies of bacterial growth laws have focused mainly on exponential growth. However, bacterial populations in the presence of high antibiotic levels can also undergo sustained exponential decay over several orders of magnitude [12, 13]. This is surprising: exponential growth can arise from deterministic cell doubling, but exponential decay with first-order kinetics typically occurs when individuals in a population die at random, like radioactive nuclei. If some cells die early while others die later, this must be due to some underlying cell-to-cell variability. The growth of single cells is known to be a stochastic, fluctuating process [14–16]. We should therefore consider the possibility that the choice between cell division and cell death could be a stochastic event. This contrasts with models that ascribe changes in exponential growth rates to changes in division rates alone [8, 9]. No analysis has so far attempted to simultaneously account for molecular fluctuations, cell division, and cell death within a common framework.

Here we show that observed macroscopic features of cell growth and decay are consistent with the hypothesis that single cells make a stochastic choice between division and death. We also show that this type of stochastic choice naturally arises from a microscopic phenomenological model of cells as collections of sub-cellular replicating units. A replicating unit is an autocatalytic set of molecules and reactions, which might include ribosomes, DNA replication initiation complexes, and metabolic loops [8, 9, 11, 17]. Such a unit is termed minimal if the removal of any of its components results in the loss of autocatalytic activity. Here we sidestep the issue of the precise composition of the minimal unit, grouping the entire replicating set of molecules and reactions into a single abstract ‘widget’.

We model the synthesis, degradation and partitioning of widgets as stochastic biochemical processes. Cell division or death occurs when a cell hits high or low thresholds of these widgets. When the average number of widgets is small, sub-cellular fluctuations in their number drive a stochastic choice between cell division and death, thus coupling molecular dynamics with cellular dynamics. Remarkably, the predictions of this basic model match observed qualitative features of cell growth and decay. These observations suggest a fourth operational definition of a minimal cell: one at the threshold of death due to irreversible loss of its last functional replicating unit.

## Methods

### Cell growth and cell density measurement protocols

We grew *Escherichia coli* MG1655 cells from a single colony overnight in Luria Bertani medium at 37 °C. We transferred 50 *μ*l of this culture to 25 ml glucose M9 minimal medium in a 100 ml flask at 37 °C. The OD_600_ of this culture was monitored until it reached 0.1. At this point, we added the appropriate concentration of kanamycin, and this was defined as the *t* = 0 time point of our measurement. Every 40 min up to a maximum of 280 min, 600 *μ*l of this culture was collected for OD_600_ measurements, and 100 *μ*l was collected for colony forming unit counts (CFU ml^−1^). We use a (non-standard) stringent definition of the minimal inhibitory concentration (MIC) as the lowest kanamycin level at which CFU ml^−1^ is non-increasing. By serially increasing [Kan] we found MIC to be between 4.2 and 4.3 *μ*g ml^−1^. Pipetting errors cause variations beyond this level of precision. The numerical value [Kan]_MIC_ = 4.21 *μ*g ml^−1^ represents the smallest increment above 4.2 *μ*g ml^−1^ at which CFU ml^−1^ was stable over 280 min. We determined OD_600_ and colony counts using multiple dilutions. Colony counts were measured for four technical replicates; at MIC we used two biological replicates, each with four technical replicates. Minimal medium (100 ml): water, 76.8 ml; 10X M9 salts 10 ml; 20% glucose, 2 ml; 1 M CaCl_2_, 10 *μ*l; 100 mM thiamine, 1 ml; 4% casamino acids, 10 ml; 1 M MgSO_4_ 200 *μ*l.

### Stochastic model of cell division and death

The transition system shown in figure 2(D) defines a Markov process with the following Master equation:

1 \begin{eqnarray*}\displaystyle \frac{{\rm{d}}{c}_{w}}{{\rm{d}}t}=-(\alpha w{c}_{w}+\gamma w{c}_{w})+(\alpha (w-1){c}_{w-1}+\gamma (w+1){c}_{w+1})\\ \,\,\,+\,2\alpha ({\rm{\Omega }}-1){c}_{{\rm{\Omega }}-1}\displaystyle \frac{1}{{2}^{{\rm{\Omega }}}-2}\left(\begin{array}{c}{\rm{\Omega }}\\ w\end{array}\right).\end{eqnarray*}

Here, each ${c}_{w}$ represents the number of cells (or the normalized probability of cells, depending on the context) with precisely *w* widgets for $w=1,\ldots ,{\rm{\Omega }}-1,$ with the stipulation that ${c}_{{\rm{\Omega }}}=0.$ The first line corresponds to cells gaining or losing individual widgets. The second line corresponds to the creation of two new daughter cells by the instantaneous division of a cell that hits ${\rm{\Omega }}$ widgets, which happens at rate $\alpha ({\rm{\Omega }}-1){c}_{{\rm{\Omega }}-1}.$ The resulting daughters are defined by $w^{\prime} $ and $w^{\prime\prime} $ such that $w^{\prime} +w^{\prime\prime} ={\rm{\Omega }}.$ The first factor of 2 accounts for two ways of achieving any given $w^{\prime} ,$ in the left or right daughter. The binomial coefficient arises since each widget has an equal chance of being inherited by either daughter cell. A cell divides instantaneously when it hits ${\rm{\Omega }}$ widgets. The usual normalizing factor of $1/{2}^{{\rm{\Omega }}}$ is replaced by $1/({2}^{{\rm{\Omega }}}-2):$ the partitions $\{w^{\prime} ,w^{\prime\prime} \}=\{0,{\rm{\Omega }}\}$ or $\{{\rm{\Omega }},0\}$ are ignored since cells repeatedly divide until some other partition occurs.

We assume a large number of total cells and widgets, so the branching process never goes extinct. If ${\boldsymbol{c}}={[{c}_{1}\ldots {c}_{{\rm{\Omega }}-1}]}^{T}$ is a column vector, the system of equations equation (1) can be written using a transition matrix $A$ and solved by matrix exponentiation:

2 \begin{eqnarray*}{\rm{d}}{\boldsymbol{c}}(t)/{\rm{d}}t=A{\boldsymbol{c}}(t)\,\,\Rightarrow \,\,{\boldsymbol{c}}(t)={{\rm{e}}}^{At}{\boldsymbol{c}}(0)\,.\end{eqnarray*}

It is convenient to write the vector ${\boldsymbol{c}}$ as a product of two components: the number of live cells ${c}_{L}=\displaystyle {\sum }_{w=1}^{{\rm{\Omega }}-1}{c}_{w},$ and the normalized distribution ${f}_{w}$ of those cells over the different numbers of widgets: ${\boldsymbol{c}}(t)\equiv {c}_{L}(t){\boldsymbol{f}}(t).$ At long times this distribution approaches the eigenvector of $A$ corresponding to its largest eigenvalue: ${\boldsymbol{f}}(t\to \infty )\to {\boldsymbol{f}}$ such that $A{\boldsymbol{f}}=\lambda {\boldsymbol{f}}.$ Therefore ${c}_{L}(t\to \infty )\sim {{\rm{e}}}^{\lambda t}.$ We can see by direct substitution that $\alpha -\gamma $ is an eigenvalue of $A.$ Since the number of live cells cannot increase any faster than the number of widgets, we also know this is its largest eigenvalue. Once ${\boldsymbol{f}}(t)$ is determined we calculate the specific division and death rates ${\phi }_{+}(t)$ and ${\phi }_{-}(t)$ as the rates at which cells cross the right boundary $w={\rm{\Omega }}-1$ and the left boundary $w=1.$ By measuring time in units of ${\alpha }^{-1},$ we can see that the values ${\phi }_{\pm }/\alpha $ depend only on the ratio $\gamma /\alpha $ and on ${\rm{\Omega }}$ (figure 4(B)).

### Probability of division

An immediate post-division daughter cell can have any widget number in the range ${w}^{{\rm{init}}}\in \{1,\,\ldots ,\,{\rm{\Omega }}-1\}.$ Starting from the initial condition ${c}_{w}^{{\rm{init}}}={\delta }_{w,{w}^{{\rm{init}}}},$ the probability of next division is a first-passage-time problem with absorbing boundaries at $w=0$ and $w={\rm{\Omega }}.$ This corresponds to a new transition matrix $\hat{A}$ where the binomial partition terms have been removed. We can find ${\boldsymbol{c}}(t)=\exp (\hat{A}t){{\boldsymbol{c}}}^{{\rm{init}}}$ and define

3 \begin{eqnarray*}{\boldsymbol{b}}=\displaystyle {\int }_{0}^{\infty }{\boldsymbol{c}}(t){\rm{d}}t={\hat{A}}^{-1}{{\boldsymbol{c}}}^{{\rm{init}}}.\end{eqnarray*}

The integrated flux leaving the right and left boundaries, corresponding to probabilities of division or death, are:

4 \begin{eqnarray*}{\wp }_{{\rm{division}}}=\alpha ({\rm{\Omega }}-1){b}_{{\rm{\Omega }}-1},\,{\wp }_{{\rm{death}}}=\gamma {b}_{1}.\end{eqnarray*}

## Results

### Measuring cell growth in the presence of antibiotics

Different classes of antibiotics act through distinct mechanisms [18]. Since we focus on replicating units, here we use the aminoglycoside antibiotic kanamycin which irreversibly binds to and inhibits the ribosome [19].

**Figure 1.**
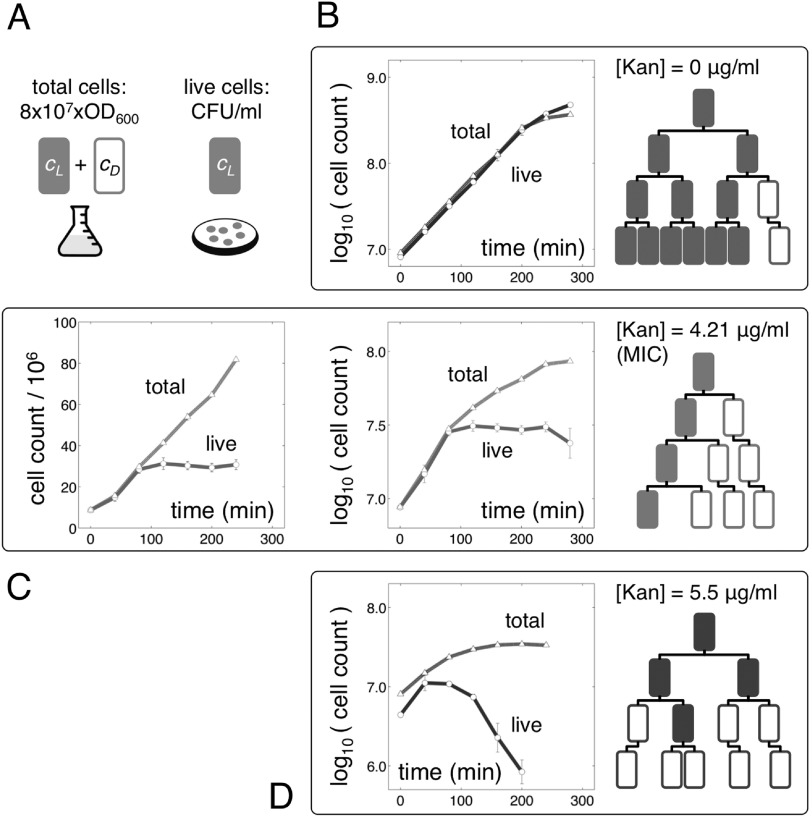
Growth and decay of cell populations under antibiotic treatment. (A) We use two methods to estimate cell densities. Turbidity (OD_600_) measures the total density of live cells (${c}_{L},$ filled rectangles) plus dead cells (${c}_{D},$ hollow rectangles). Colony forming units (CFU ml^−1^) measures the density of live cells (${c}_{L}$) alone. We always report the rescaled value 8 × 10^7^ × OD_600_ which can be directly compared to CFU ml^−1^. (B)–(D) Cell growth measurements for *Escherichia coli* populations. Each panel corresponds to different concentrations of the antibiotic kanamycin. Light curves, triangle symbols: OD_600_ measuring total cells. Dark curves, circle symbols: CFU ml^−1^ measuring live cells. Errorbars show standard deviations over replicates. Schematics on the right show examples of cell division and death that would produce such growth curves. (B) At zero antibiotic the total cell and live cell numbers completely overlap and show perfect exponential growth on a log–lin plot; we have inserted an offset so both can be seen. Schematically: cell division dominates and cell death is negligible, driving exponential growth each generation. (C) At the MIC of antibiotic live cell number flattens out while total cell number continues to increase linearly on a lin–lin plot (left). Schematically: each time a cell divides, on average one of its daughters dies while the other goes on to a next successful division. Thus the live cell number is constant while the dead cell number increases linearly each generation. (D) At high antibiotic, total cell number flattens out while live cell number decays exponentially on a log–lin plot. Schematically: though cells transiently go through a few rounds of division, eventually all cells die so live cell number goes to zero while total cell number reaches a constant limit.

The effect of an antibiotic is typically quantified in terms of its impact on growth rates. It is common to use turbidity measurements (OD_600_) for this purpose, since these are easy to perform and automate [20]. However, cell growth is more accurately determined by measuring the density of viable colony-forming units (CFU ml^−1^) [13]. These two are not equivalent: colony-forming units measure the density of live cells, whereas turbidity measures the total density of all non-lysed cells, live or dead (figure 1(A)). For the remainder of our analysis we always compare CFU ml^−1^ (live cells) with the rescaled value 8 × 10^7^ × OD_600_ (total cells) as these coincide for exponentially growing *E. coli.* cells in the absence of antibiotics. Since we use a fixed volume of media we use the terms cell number and cell density interchangeably.

The MIC of an antibiotic is often defined as the concentration at which OD_600_ no longer increases, but this depends on the duration and sensitivity of the measurement [21]. Here we rigorously define MIC as the antibiotic concentration at which CFU ml^−1 ^is asymptotically constant over time.

### Live and total cell counts under antibiotic treatment

We monitored the effect of kanamycin addition on *E. coli* cells grown in an initially antibiotic-free medium (figure 1; Methods: Cell growth and cell density measurement protocols). To get a detailed picture of the effect of the antibiotic, we simultaneously measured CFU ml^−1^ (live cells) and OD_600_ (total cells) over time. After a brief transient, CFU ml^−1^ settled into an exponentially growing profile (for low [Kan]; figure 1(B)) or an exponentially decaying profile (for high [Kan]; figure 1(D)). At the boundary between these two regimes CFU ml^−1^ remained constant over time, defining the MIC ([Kan] = 4.21 *μ*g ml^−1^; figure 1(C)). The behavior of turbidity was strikingly different: OD_600_ always monotonically increased, with an exponentially accelerating profile (for low [Kan]; figure 1(B)) or a concave decelerating profile (for high [Kan]; figure 1(D)). At the boundary between these two regimes, OD_600_ increased precisely linearly (figure 1(C), left panel). These trends persisted until the medium was depleted of nutrients. Together, these observations are consistent with the idea that CFU ml^−1^ measures the density of live cells (${c}_{L},$ which can increase or decrease) while OD_600_ measures the total density of live plus dead cells (${c}_{L}+{c}_{D},$ which can only increase). This interpretation assumes a low rate of cell lysis. We have also not considered persister cells that slow their division under antibiotic treatment [22]; these are significant once nearly all cells in the original population have already died.

### Exponential growth or decay arises from first-order cell division and death rates

**Figure 2.**
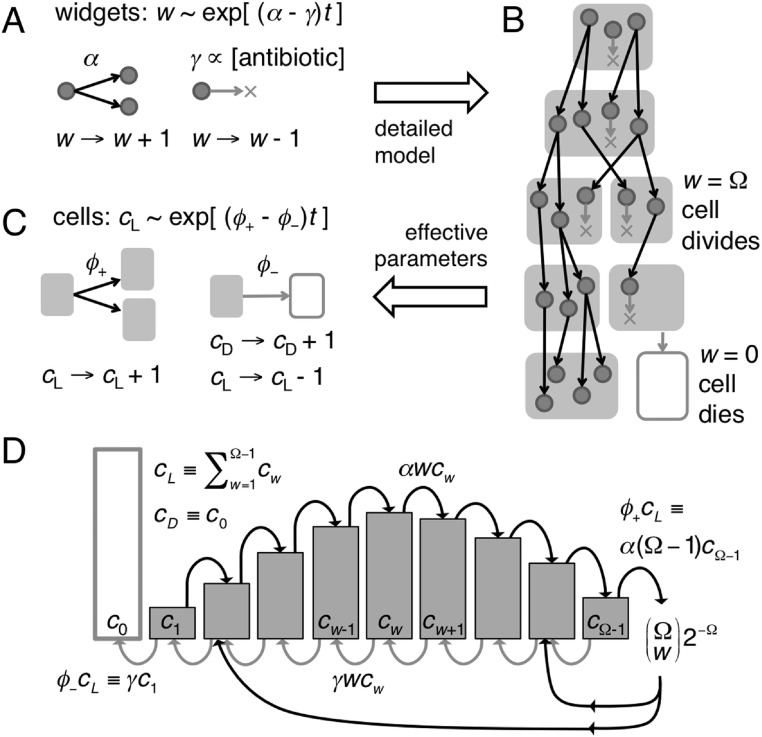
A stochastic model of cell division and death. (A) A widget is a minimal replicating unit obeying a birth-death process with rates $\alpha $ and $\gamma ,$ the latter proportional to antibiotic levels. (B) Cells are collections of widgets. When a cell hits *w* = Ω it divides; when it hits *w* = 0 it dies. (C) The widget dynamics can be used to define the cell dynamics, with effective time-dependent division and death rates *ϕ*_±_. (D) Dynamics of a cell population: ${c}_{w}$ represents the number of cells with exactly $w$ widgets. Individual cells move to the right (gain a widget) or left (lose a widget). *ϕ*_−_ is the per-cell rate at which cells cross the left boundary at *w* = 1 and die. ${\phi }_{+}$ is the per-cell rate at which cells cross the right boundary at *w* = Ω − 1 and divide. At division the widgets binomially partition into two daughter cells, which re-enter the main distribution. The area of the red bin gives the number of dead cells, the total area of the gray bins gives the number of live cells. Over time the gray distribution reaches a constant shape but can increase or decrease in area.

As a first attempt to understand these dynamics, we decomposed the separate contributions of cell division (${\phi }_{+}$) and death (${\phi }_{-}$) rates using a first-order kinetic model (figure 2(C)):

5 \begin{eqnarray*}{\rm{d}}{c}_{L}/{\rm{d}}t=({\phi }_{+}-{\phi }_{-}){c}_{L},\\ {\rm{d}}{c}_{D}/{\rm{d}}t={\phi }_{-}{c}_{L}.\end{eqnarray*}

If there are no constraints on ${\phi }_{\pm },$ equation (5) is a tautology. The first-order kinetics assumption implies that, once the transient response to the antibiotic has settled but nutrients are not yet depleted, ${\phi }_{\pm }$ are constant over time. The solution to equation (5) is then:

6 \begin{eqnarray*}\displaystyle \frac{{c}_{L}}{{c}_{L}^{{\rm{init}}}}={{\rm{e}}}^{({\phi }_{+}-{\phi }_{-})t},\\ \displaystyle \frac{{c}_{D}}{{c}_{L}^{{\rm{init}}}}=\displaystyle \frac{{c}_{D}^{{\rm{init}}}}{{c}_{L}^{{\rm{init}}}}+\displaystyle \frac{{\phi }_{-}}{{\phi }_{+}-{\phi }_{-}}({{\rm{e}}}^{({\phi }_{+}-{\phi }_{-})t}-1),\end{eqnarray*}

where we have scaled cell numbers by their initial values at the end of the transient.

${\phi }_{\pm }$ depend on the antibiotic concentration, three values of which are of particular interest. At zero antibiotic we expect ${\phi }_{+}\equiv {\phi }_{+}^{{\rm{\max }}}$ and ${\phi }_{-}=0,$ so ${c}_{L}={c}_{L}^{{\rm{init}}}{{\rm{e}}}^{{\phi }_{+}^{{\rm{\max }}}t}$ and ${c}_{D}=0.$ Indeed, we see that total cell number and live cell number are equal, and both grow with exponent ${\phi }_{+}^{{\rm{\max }}}=0.017\,$ min^−1^ (figure 1(B)). At high antibiotic we expect ${\phi }_{+}=0$ and ${\phi }_{-}\equiv {\phi }_{-}^{{\rm{\max }}},$ so ${c}_{L}={c}_{L}^{{\rm{init}}}{{\rm{e}}}^{-{\phi }_{-}^{{\rm{\max }}}t}$ and ${c}_{D}={c}_{L}^{{\rm{init}}}-{c}_{L}.$ This again matches the data: we see that live cell number decays exponentially, while total cell number approaches a flat asymptote (figure 1(D)).

At MIC the situation is more interesting since by definition ${\phi }_{+}={\phi }_{-}\equiv {\phi }^{{\rm{MIC}}},$ so ${c}_{L}(t)={c}_{L}^{{\rm{init}}}$ and ${c}_{D}(t)={c}_{D}^{{\rm{init}}}+{c}_{L}^{{\rm{init}}}{\phi }^{{\rm{MIC}}}t.$ That is, live cell numbers are constant because death and division rates balance, while dead cell numbers increase linearly because they arise from the continuing death of live cells. This is precisely what we observe: the slope of the linear portion of the total cell curve at MIC shows that ${\phi }^{{\rm{MIC}}}=0.011$ min^−1^ (figure 1(C), left panel). The total cell curve tracks the live cell curve for the first hour following antibiotic treatment, after which the live cell curve flattens while the total curve increases linearly. This suggests cell death only begins after a lag, while cell division is relatively unperturbed by the antibiotic. This is corroborated by the ratio ${\phi }^{{\rm{MIC}}}/{\phi }_{+}^{{\rm{\max }}}\sim 0.6$ being close to unity: cell division is nearly as rapid at MIC as at zero antibiotic.

In summary, the following three observations support the idea that cell division and death operate at the single-cell level with apparent first-order kinetics, once transients die out. At low antibiotic the growth of total cell number and live cell number are both exponential. At high antibiotic live cell number decays exponentially, while total cell number approaches a constant. In between, at MIC, total cell number increases linearly, while live cell number is constant. Seeing first-order kinetics across the full range of antibiotic concentrations is surprising, since cells are not elementary chemical entities. In the following section we show how such kinetics emerge from a stochastic model of a cell as a collection of minimal replicating units.

### Widgets: sub-cellular replicating units

The phenomenological model of equation (5) fails to predict the transient dynamics because it assumes a cell has no internal structure. If we wish to determine how ${\phi }_{\pm }$ depend on time, this must either be directly measured, or predicted from a more microscopic model. We therefore consider a cell as a collection of replicating units we term ‘widgets’ (figure 2; Methods: Stochastic model of cell division and death). The widgets themselves obey a birth-death dynamics analogous to equation (5), but with microscopic birth and death rate constants $\alpha $ and $\gamma $ (figure 2(A)). For concreteness we imagine $\alpha $ to be constant (e.g. the catalytic efficiency of ribosomes) while $\gamma $ depends on the antibiotic concentration (e.g. the rate of irreversible ribosome inhibition by kanamycin), but these assumptions may be relaxed. We specify how cell division and death depend on the widgets as follows (figure 2(B)). When the widget number hits $w=0$ the cell dies since no new widgets can be made without an existing widget. When the widget number hits a threshold $w={\rm{\Omega }}$ the cell instantaneously divides, and the widgets are partitioned binomially between two daughter cells. This is arguably the simplest possible microscopic model of cell growth.

We consider a population of cells, binned according to the number of widgets they contain: ${c}_{w}$ is the number of cells with precisely $w$ widgets, for $w=1,\,\ldots ,\,{\rm{\Omega }}-1$ (figure 2(D); Methods: Stochastic model of cell division and death). Individual cells do a biased Poisson random walk along the $w$-axis: moving to the right if they gain a widget, or to the left if they lose one. The number of live and dead cells are:

7 \begin{eqnarray*}{c}_{L}=\displaystyle \sum _{w=1}^{{\rm{\Omega }}-1}{c}_{w}\,{c}_{D}={c}_{0}.\end{eqnarray*}

Cells that cross the left boundary ($i=1$) move to bin ${c}_{0}$ and die, so ${c}_{L}$ decreases by one and ${c}_{D}$ increases by one. Cells that cross the right boundary ($i={\rm{\Omega }}-1$) move to the bin ${c}_{{\rm{\Omega }}}$ and instantaneously divide into two daughters, so ${c}_{L}$ increases by one. The daughters re-enter the distribution at two positions $w^{\prime} $ and $w^{\prime\prime} $ such that ${w}^{\prime} +{w^{\prime\prime}}={\Omega},$ with $w^{\prime} $ binomially distributed. These processes define a transition matrix $A$ (Methods: Stochastic model of cell division and death) so:

8 \begin{eqnarray*}{\rm{d}}{c}_{v}(t)/{\rm{d}}t=\displaystyle \sum _{w=1}^{{\rm{\Omega }}-1}{A}_{vw}(\alpha ,\gamma ){c}_{w}(t).\end{eqnarray*}

This can be easily solved for ${c}_{w}(t)$ from any ${c}_{w}(t=0)$ by matrix exponentiation. Over time the distribution of live cells over widget number reaches a steady-state, proportional to the eigenvector of the transition matrix corresponding to its largest magnitude eigenvalue $\lambda .$ If we define a normalized distribution ${f}_{w}(\alpha ,\gamma )$ such that $A{\boldsymbol{f}}=\lambda {\boldsymbol{f}},$ then:

9 \begin{eqnarray*}{c}_{w}(t\to \infty )={c}_{L}(t){f}_{w}\sim {{\rm{e}}}^{\lambda t}{f}_{w}.\end{eqnarray*}

That is, the shape of the distribution becomes constant, while the total number of cells increases or decreases exponentially.

### Comparison of widget model to experimental growth curves

If we measure time in units of ${\alpha }^{-1}$ the model has two dimensionless parameters: the widget death/birth ratio $\gamma /\alpha ,$ and the threshold number of widgets at cell division ${\rm{\Omega }}.$ The value of $\gamma /\alpha $ is some monotonically increasing function of antibiotic concentration, not necessarily linear, with $\gamma /\alpha =1$ at MIC. We are left with a single tunable parameter ${\rm{\Omega }}$ which controls the number of widgets and therefore influences the scale of stochastic fluctuations: higher values of ${\rm{\Omega }}$ correspond to lower fluctuations relative to the mean. We will return to this point in our discussion.

**Figure 3.**
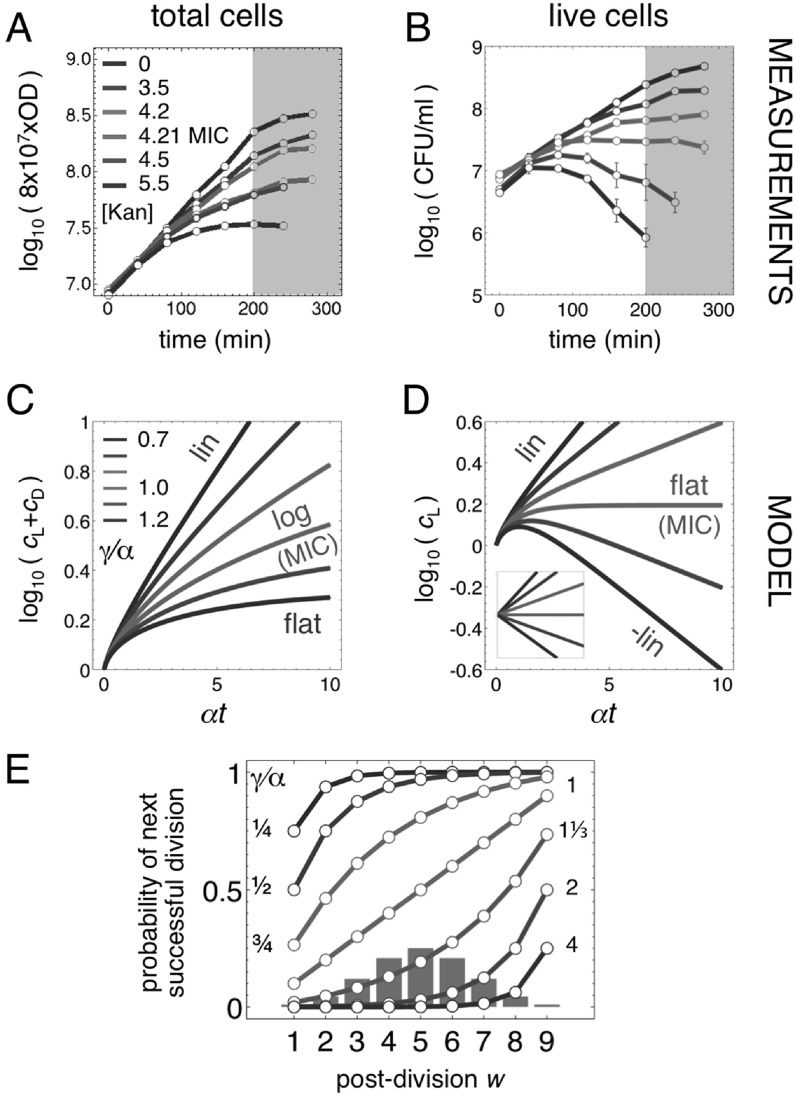
Comparison of widget model to experimental growth curves. (A), (B) *E. coli* growth curves. We show the same data as in figure 1, including additional antibiotic levels. Each curve corresponds to different concentrations of kanamycin; see key in panel (A). [Kan] = 4.21* μ*g ml^−1^ is the minimal inhibitory concentration (MIC) at which live cell number is constant over time. The gray area indicates the nutrient depletion zone where exponential growth stalls. (A) Total cell number (OD_600_) over time, mean and standard deviation over four technical replicates. (B) Live cell number (CFU ml^−1^) over time, mean and standard deviation over four technical replicates. (C), (D) Predictions of the widget model. Curves show solutions to equation (8) for the division threshold ${\rm{\Omega }}=10$ and increasing values of $\gamma /\alpha ,$ corresponding to increasing antibiotic levels; see key in panel (C). $\gamma /\alpha =1$ corresponds to MIC. We label the asympotic behavior of the predicted curves: linear (lin), logarithmic (log), or flat. (C) Total cell number ${c}_{L}+{c}_{D}.$ (D) Live cell number ${c}_{L}.$ The inset shows the prediction for a cell that has no internal structure and divides as soon as ${\rm{\Omega }}=2;$ no transient is observed in this case. (E) Two sources of fluctuations: random partitioning and random birth/death of widgets. Immediately after cell division, the number of widgets $w$ in a daughter cell is binomially distributed (gray histogram). Starting at any widget number, random birth/death dynamics can take a cell to either boundary. We show the probability that a cell will successfully divide again rather than die (curves; colors represent different values of $\gamma /\alpha $ for ${\rm{\Omega }}=10$).

In our experiments we first grow cells in in the absence of antibiotic and then add kanamycin at the initial measurement point. To model this we first find the stationary distribution of widgets at zero antibiotic, and set this as the initial condition: ${f}_{w}(\alpha =1,\gamma =0).$ The addition of antibiotic is modeled by shifting $\gamma $ to some non-zero value, causing the cell population to evolve toward a new asymptotic distribution: ${f}_{w}(\alpha =1,\gamma ).$ This corresponds to the transient phase of the experiment, as cells adapt to the presence of the antibiotic. As cells go through this transient we can use equation (8) to find ${c}_{w}(t)$ and equation (7) to find ${c}_{L}(t)$ and ${c}_{D}(t)$ for various ratios $\gamma /\alpha .$ The transient lasts longer if $\gamma /\alpha $ is low or if ${\rm{\Omega }}$ is high. Its duration is essentially determined by the inverse of the second-largest eigenvalue of $A;$ ${\rm{\Omega }}=2$ is a singular case where there is no transient (see figure 3(D) inset).

The predictions of our model for ${\rm{\Omega }}=10$ (figures 3(C), (D)) qualitatively match our experimental observations (figures 3(A), (B)). In particular, we capture the initial transient increase in cell number as cells adjust to the addition of antibiotic. We correctly predict the asymptotic exponential growth and decay kinetics of live cell number at low or high antibiotic levels (figures 3(B), (D)). Finally, we correctly predict the response at MIC, where the live cell number flattens (figures 3(B), (D)) while total cell number approaches a linear trajectory (figures 1(C), 3(A), (C)).

Note that the assignment ${\rm{\Omega }}=10$ is not a numerical fit, it is a representative parameter choice. It is not justifiable to fit the abstract widget model to quantitative measurements of cell growth, which are expected to depend on more complex aspects of metabolism and cell size control [11]. Nevertheless it is remarkable that such a basic model captures diverse qualitative aspects of cell growth and decay across a range of antibiotic concentrations.

### Widget fluctuations drive a stochastic choice between cell division and death

A key aspect of the model is the choice of a post-division cell between two ultimate fates: division and death (Methods: Probability of division). Immediately following division the widgets partition binomially between daughter cells, leading to an initial post-division variation (histogram, figure 3(E)). At this point, the fluctuating birth-death widget dynamics take over. Cells with an initially low value of $w$ are more likely to hit the left boundary and die, while cells with an initially high value are more likely to hit the right boundary and divide (curves, figure 3(E)). The addition of antibiotics (increasing $\gamma /\alpha $) biases the choice against division. The squared coefficient of variation of the post-division binomial distribution $1/{\rm{\Omega }}$ is a convenient measure of fluctuations. Note that increasing $1/{\rm{\Omega }}$ increases fluctuations in both binomial widget partitioning and Poisson birth/death dynamics. The fewer the number of widgets, the larger the scale of fluctuations relative to the mean.

### Deriving cellular parameters from widget properties

Having validated the model against a specific set of experiments, we now use it to predict aspects of cell growth over a broader range of conditions. The dynamics of the widgets can be used to determine the effective parameters ${\phi }_{\pm }$ that appear in equation (5) (figure 2(D)). To do this we first find the number of cells in each widget bin, then track how many cells cross the right or left boundary. We can thus write an equation similar to equation (5), where ${\phi }_{\pm }$ are now time-dependent because the distribution of cells evolves from its initial state:

10 \begin{eqnarray*}{\phi }_{+}(t)=\alpha ({\rm{\Omega }}-1){c}_{{\rm{\Omega }}-1}(t)/{c}_{L}(t),\\ {\phi }_{-}(t)=\gamma {c}_{1}(t)/{c}_{L}(t).\end{eqnarray*}

Equations (9) and (10) imply that ${\phi }_{\pm }$ eventually become time-independent first-order rates.

Classically, exponentially growing populations are thought to arise when post-division daughter cells reach a time-invariant composition [5]. Our analysis suggests a broader pattern in which exponentially growing or decaying populations arise because the entire population of cells reaches a time-invariant distribution over compositions, due to which the per-cell rates of division and death appear to be first-order constants. However, these constants themselves obey certain constraints. Comparing equations (9) to (6) we have $\lambda ={\phi }_{+}-{\phi }_{-}.$ On the other hand, it is easy to check that the largest eigenvalue of $A$ is given by $\lambda =\alpha -\gamma $ (Methods: Stochastic model of cell division and death). This gives us two completely distinct ways to decompose the growth exponent $\lambda $ in the limit $t\to \infty :$

11 \begin{eqnarray*}\lambda =\alpha -\gamma ={\phi }_{+}-{\phi }_{-}.\end{eqnarray*}

This key equation relates cellular parameters to molecular parameters. It shows that cell division and death rates are fundamentally constrained and coupled by widget birth and death rates.

### Cell division and death rates at MIC: cell stasis as a low-fluctuation limit

**Figure 4.**
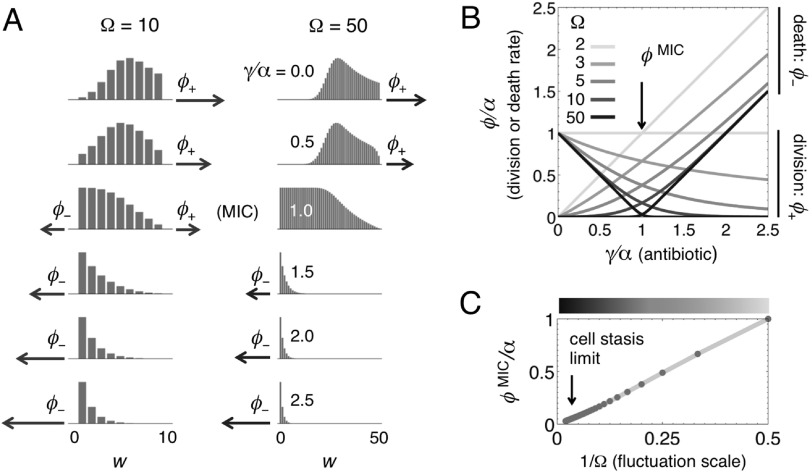
Stochastic cell division and cell death. (A) Once sufficient time has passed, distributions of cells over widget number reach a constant shape ${f}_{w},$ as in equation (9). We show widget distributions (gray histograms, scaled to fixed height) as $\gamma /\alpha $ is increased (top to bottom) for two different values of ${\rm{\Omega }}$ (left and right). $\gamma /\alpha =1$ corresponds to MIC; low ${\rm{\Omega }}$ is high fluctuations, high ${\rm{\Omega }}$ is low fluctuations. Maroon arrows show the resulting rates of cell division (${\phi }_{+}$) and cell death (${\phi }_{-}$). (B) Division rate (${\phi }_{+};$ decreasing curves) and death rate (${\phi }_{-};$ increasing curves) as a function of antibiotic level ($\gamma /\alpha $) for various values of ${\rm{\Omega }}.$ Darker curves (higher ${\rm{\Omega }}$) correspond to smaller fluctuations. MIC is defined by the point at which ${\phi }_{+}={\phi }_{-}\equiv {\phi }^{{\rm{M}}{\rm{I}}{\rm{C}}}.$ (C) 1/${\rm{\Omega }}$ is the squared coefficient of variation of the post-division binomial distribution of widgets, and is a convenient measure of fluctuations. As the scale of fluctuations decreases ${\phi }^{{\rm{M}}{\rm{I}}{\rm{C}}}$ drops, ultimately reaching the classic cell stasis limit of zero division and death.

There are many ways to split ${\phi }_{\pm }$ so the constraint of equation (11) is satisfied. In general the division rate (${\phi }_{+}$) decreases and the death rate (${\phi }_{-}$) increases with antibiotic level, but the form of these curves depends crucially on the scale of fluctuations ($1/{\rm{\Omega }}$). At zero antibiotic $\gamma =0$ so ${\phi }_{+}\equiv {\phi }_{+}^{{\rm{\max }}}=\alpha ,$ and ${\phi }_{-}=0.$ The question is more interesting at MIC, where $\alpha =\gamma $ so ${\phi }_{+}={\phi }_{-}\equiv {\phi }^{{\rm{MIC}}}.$ In this scenario cell division and death rates must be equal, but it is not obvious how large each rate is individually. To calculate this we track the steady-state distribution of cells over widget number (figure 4(A)). The more cells at the right boundary, the greater the rate of division; the more at the left boundary, the greater the rate of death. Addition of antibiotics shifts the distribution leftward, increasing the death rate (figure 4(A), top to bottom). More interestingly, decreasing the scale of fluctuations (increasing ${\rm{\Omega }}$) narrows the distribution away from the boundaries and decreases both division and death rates while keeping $\alpha -\gamma $ constant (compare figure 4(A), left and right columns).

When we plot how ${\phi }_{+}$ and ${\phi }_{-}$ vary with $\gamma /\alpha $ we see a range of behaviors depending on the value of ${\rm{\Omega }}$ (figure 4(B)). In the low-fluctuation limit of high ${\rm{\Omega }}$ (darkest curves) the cell division and death curves collapse on to two diagonal lines. This corresponds to the textbook scenario of bacterial dynamics: below MIC, exponential growth is driven purely by cell division with zero cell death; above MIC exponential decay is driven purely by cell death with zero cell division; and at MIC we have ${\phi }^{{\rm{MIC}}}=0$ so cells neither divide nor die. However, we now see that this classic picture of cell stasis under antibiotic treatment is the low-fluctuation limit of a more general dynamics. As fluctuations are increased by decreasing ${\rm{\Omega }}$ (lighter curves), cell division and death curves are both pushed higher, thus ${\phi }^{{\rm{MIC}}}$ also increases. In the high-fluctuation limit ${\rm{\Omega }}=2$ (lightest curves) each cell contains only one widget, and instantaneously divides as soon as this widget replicates. Cell dynamics are thus identical to widget birth/death dynamics, so ${\phi }_{+}=\alpha ,$ ${\phi }_{-}=\gamma ,$ and ${\phi }^{{\rm{MIC}}}$ is high. Overall we see that ${\phi }^{{\rm{MIC}}}$ scales in proportion $1/{\rm{\Omega }}$ (figure 4(C)), demonstrating that cell division and death at MIC are fundamentally driven by fluctuations in widget number.

## Discussion and conclusion

### Biological implications of stochastic cell division and death

The very existence of minimal units of replication implies that microscopic division and death continue while the macroscopic count of live cells remains static. In terms of therapy this is relevant since each dead cell raises the possibility of sepsis in an infection context. Each cell division is also coupled to a DNA replication event, and represents a chance for new mutations to arise. Fluctuation-induced divisions therefore indirectly increase the possibility of antibiotic resistance. There are additional implications for bacterial survival under stress. Suppose we tracked the fate of a single dividing cell in the presence of antibiotics. Stochastic partitioning of molecules is a form of asymmetric cell division, so the two daughters could take on different fates. In our simple model daughters are anti-correlated in their probability of subsequent division: the one that passively inherits more widgets has the higher probability. We could imagine alternative models in which cells under stress preferentially partition functional components to one daughter, thus enhancing survival rates. Cell-to-cell variability has been reported in the response to sub-lethal antibiotic levels [23]. Asymmetric partitioning has also been implicated in bacterial ageing [24]. Single-cell experiments could be used to track the probability of next division of immediate post-division cells. Correlations or anti-correlations in this probability between two daughters would reveal more complex partitioning than we have considered here.

### The molecular nature of a widget

Measurements of an invariant cell initiation mass across a wide range of growth conditions [11] suggest that a bacterial cell comprises only a handful of minimal replication units, ranging from about two to eight. Our approach based on stochastic division and death also suggests that there are only a few minimal replication units per cell: in figure 3 we show that the model matches qualitative aspects of our cell growth curves for ${\rm{\Omega }}=10.$ For this value of ${\rm{\Omega }}$ we would predict the rate of division and death at MIC to be ${\phi }^{{\rm{MIC}}}=0.17\times {\phi }_{+}^{{\rm{\max }}}$ (figure 4(B)) whereas the measured value is much higher at ${\phi }^{{\rm{MIC}}}=0.6\times {\phi }_{+}^{{\rm{\max }}}.$ This suggests either an even lower value of ${\rm{\Omega }}$ or, more likely, additional sources of fluctuations in real cells. To account for this one could construct a model in which the division trigger and fluctuation sources were decoupled.

The low inferred number of replicating units per cell immediately shows that a widget is not a single molecular complex such as a ribosome, but rather a collection of diverse molecules and reactions. However, our approach does not give further clues about the nature of the replicating unit. We can imagine extending our model to more realistic cellular compositions, beyond collections of identical widgets. Such a model would specify the manifolds corresponding to compositional states that trigger cell division or death. Given enough time we would expect any initial population of cells to reach a steady-state distribution of compositions, from which first-order division or death rates can be calculated. For each point in composition space we could calculate the probability of successful next division. In this way we separate the space into regions that have zero division probability, or non-zero division probability. In practice we might define the locus of points at which the division probability drops to some low level, say 1%. The fact that a cell recovers at all from such bleak initial conditions means it must contain at least one minimal replicating unit. If it is feasible to experimentally isolate and characterize cells along this locus, we would arrive at a new operational definition of a minimal cell: one at the very boundary of death. Monod [25] might have been pleased with such a definition.
